# Internal and External Dispersal of Plants by Animals: An Aquatic Perspective on Alien Interference

**DOI:** 10.3389/fpls.2018.00153

**Published:** 2018-02-13

**Authors:** Casper H. A. van Leeuwen

**Affiliations:** Department of Aquatic Ecology, Netherlands Institute of Ecology (NIOO-KNAW), Wageningen, Netherlands

**Keywords:** ectozoochory, endozoochory, exotic, frugivore, invasive, mutualism, non-native, seed

## Abstract

Many alien plants use animal vectors for dispersal of their diaspores (zoochory). If alien plants interact with native disperser animals, this can interfere with animal-mediated dispersal of native diaspores. Interference by alien species is known for frugivorous animals dispersing fruits of terrestrial plants by ingestion, transport and egestion (endozoochory). However, less attention has been paid to possible interference of alien plants with dispersal of diaspores via external attachment (ectozoochory, epizoochory or exozoochory), interference in aquatic ecosystems, or positive effects of alien plants on dispersal of native plants. This literature study addresses the following hypotheses: (1) alien plants may interfere with both internal and external animal-mediated dispersal of native diaspores; (2) interference also occurs in aquatic ecosystems; (3) interference of alien plants can have both negative and positive effects on native plants. The studied literature revealed that alien species can comprise large proportions of both internally and externally transported diaspores. Because animals have limited space for ingested and adhering diaspores, alien species affect both internal and external transport of native diaspores. Alien plant species also form large proportions of all dispersed diaspores in aquatic systems and interfere with dispersal of native aquatic plants. Alien interference can be either negative (e.g., through competition with native plants) or positive (e.g., increased abundance of native dispersers, changed disperser behavior or attracting additional disperser species). I propose many future research directions, because understanding whether alien plant species disrupt or facilitate animal-mediated dispersal of native plants is crucial for targeted conservation of invaded (aquatic) plant communities.

## Introduction

Dispersal of plant diaspores by animals (zoochory) is a globally important mechanism regulating species diversity of plant communities. Key disperser species include birds, mammals, and fish–of which many forage on plant diaspores, transport them internally during digestion, and defecate viable diaspores in new locations (endozoochory, Horn et al., 2011; Van Leeuwen et al., 2012; Albert et al., 2015; Corlett, 2017). Plant diaspores are also transported externally on animals by adhering to their feathers, fur or feet (referred to as ectozoochory, epizoochory or exozoochory; Sorensen, 1986; Will et al., 2007; Coughlan et al., 2017). Zoochory can provide long-distance transport over several hundreds of kilometers (Viana et al., 2016), but is also important at local scales (Kleyheeg et al., 2017). Morphological adaptations of diaspores for zoochory are therefore abundant in both temperate (35–60% of all terrestrial plant species) and tropical (75–90% of all woody plant species) regions (Willson et al., 1990; Jordano, 2000; Herrera, 2003).

Humans also transport many plant diaspores across the world, which frequently introduces alien species into networks of native plants and animals (Ricciardi, 2007; Hulme, 2015). Alien species can comprise large proportions of all species in ecosystems (Pyšek, 1998). Arrival of alien species in native communities can have numerous ecological and evolutionary consequences, notably if alien species naturalize and start new interactions with native species (Richardson et al., 2000a; Vilà et al., 2011; Dickie et al., 2017). These new species interactions between alien and native species can alter the interactions that were previously present among only native species (Traveset et al., 2015). For example, insects pollinating native flowers can start to prefer flowers of alien plants (Gibson et al., 2013), or birds previously foraging on native seeds can start ingesting and dispersing mainly alien seeds (Heleno et al., 2013). Recent reviews have convincingly shown this potential of alien species to interfere with native species interactions (Traveset and Richardson, 2006, 2011, 2014), and the implications of this for conservation (Buckley et al., 2006).

Most studies to date, however, investigated this phenomenon for internally transported terrestrial plant seeds and fruits. The aim of this literature study is to explore the potential of alien species to interfere with external transport of plant diaspores, to examine potential interference specifically in aquatic ecosystems, and to evaluate whether alien species commonly have negative or positive effects on native plant species. I therefore address the following three hypotheses: (1) alien plant species may interfere with both internal and external animal-mediated dispersal of native diaspores; (2) interference by alien plants also commonly occurs in aquatic ecosystems; and (3) interference by alien plants can have both negative and positive effects on native plants. I focus on zoochory of plant diaspores (mostly fruits and seeds, here respectively considered the fleshy parts and hard structures of reproductive units) by vertebrate animals. “Alien species” is used as the terminology throughout this study to refer to all non-native, invasive, introduced or exotic species that established a self-sustaining reproductive population outside their native geographic area (naturalized sensu Richardson et al., 2000b).

## Effects of alien plants on endo- and ectozoochory

Interference of alien diaspores is best known for endozoochory. Common interference involves disperser animals ingesting alien plant diaspores instead of native diaspores (Traveset and Richardson, 2006, 2011, 2014). The level of interference by alien species therefore relies strongly on their relative attractiveness to foraging disperser species. Most key disperser species for endozoochory—such as frugivorous birds (Jordano, 2000), waterbirds (Reynolds et al., 2015; Green, 2016), large mammals (Albert et al., 2015), and fish (Horn et al., 2011)—forage highly selectively on the richest food sources (Pyke et al., 1977). Diaspore ingestion therefore strongly relies on diaspore traits like attractive coloring, high nutrient content, small or large size, high sugar content, or long fruiting periods (e.g., Levey and Del Rio, 2001; Gosper et al., 2005; Westcott and Fletcher, 2011). Hence, especially attractive alien species may become incorporated into the diets of native animals, cause interference, and have great potential to disrupt endozoochory as a dispersal mechanism.

Interference with ectozoochory may work analogously, but has been little investigated. Ectozoochory by vertebrates is most common for diaspores that use hooks, mud or mucus to attach to fur, hooves, feathers or feet (Sorensen, 1986; Van Leeuwen and Van Der Velde, 2012; Schulze et al., 2014; Reynolds and Cumming, 2016; Coughlan et al., 2017). Because space on animals for diaspore attachment is limited, animals that already carry many diaspores will have less space available for attachment of native diaspores. This implies that also for externally dispersed diaspores, alien plant species may interfere with dispersal of native plant species. However, to my knowledge, no studies have specifically addressed this idea.

The impact of interference likely differs between native diaspores relying on either endo- or ectozoochory, which can be better understood by looking at the evolutionary histories of both dispersal mechanisms. While endozoochory is mostly a mutualistic interaction that benefits both the plant and animal, ectozoochory is commonly only beneficial for plants and has little or no direct effects on animals. Diaspore morphology is important for dispersal in the case of both dispersal mechanisms—for instance for survival during transport—but only for endozoochory the uptake phase in the dispersal process involves active diaspore selection by foraging animals. For endozoochory, examples of disperser animals preferring alien diaspores over native diaspores are common: Canada geese (*Branta canadensis*) feed selectively on Eurasian grasses in Canada (Best and Arcese, 2009), various native South African bird species disproportionately feed on an alien shrub with very abundant and nutritious fruits (Mokotjomela et al., 2013b,c), and Black-tailed jackrabbits (*Lepus californicus*) and mule deers (*Odocoileus hemionus*) remove fruits of an alien succulent faster than those of a native species in the same genus (Vilà and D'Antonio, 1998). This implies that active selection by disperser animals plays an important role in alien interference, at least in the case of endozoochory. For ectozoochory, active diaspore selection is lacking. This puts forward the idea that alien species may have a stronger potential to interfere with endozoochory than ectozoochory, which should be further studied.

This section indicated that alien interference with ectozoochory is plausible, but may be less disruptive than in cases of endozoochory. It mostly indicated, however, that interference with ectozoochory is a hardly studied phenomenon that warrants investigation. Key directions for future research therefore include (1) studying interference of alien plants with ectozoochory by itself, and simultaneously with interference of endozoochory in single model systems; and (2) comparing the relative effectiveness of alien and native diaspores for endo- and ectozoochory in choice and attachment experiments (see e.g., Greenberg et al., 2001; Gosper et al., 2006; Mokotjomela et al., 2013a,b). We can expect highly attractive alien diaspores, those with high similarity to native diaspores (Gosper and Vivian-Smith, 2009), or with disproportionally strong attachment capabilities (Will et al., 2007; Collas et al., 2014) to cause greater interference with zoochory. (3) Lastly, alien impact can strongly change over time since invasion (e.g., Strayer et al., 2017), which could be used to detect evolutionary responses in diaspore traits of native species. This would require comparative studies on systems that have been invaded at different moments in the past, or could be analyzed by using historic data.

## Effects of alien plant species on zoochory in aquatic systems

The existence of several recent reviews on alien interference with endozoochory of terrestrial plant species (Traveset and Richardson, 2006, 2011, 2014)—and the lack thereof for aquatic ecology—suggests that interference is mostly a concern for the conservation of terrestrial ecosystems. This section explores whether interference has also been documented in aquatic habitats, because if so, it could have analogous consequences for aquatic ecosystems and provide interesting direction for future (aquatic) research.

To compare study effort on alien interference between habitats and among disperser animals, I performed a standardized search for publications reporting the presence of alien and native aquatic diaspores in single samples of feces or attached to native vertebrate animals in ISI Web of Science. On the 11th of January 2018 I entered the following search string: TS = (alien OR exotic OR non-native OR invas^*^ OR introduce^*^) AND TS = (seed OR diaspore^*^ OR fruit OR nut); combined with either TS = (frugivor^*^ OR endozoochor^*^) or with TS = (ectozoochor^*^ OR epizoochor^*^ OR exozoochor^*^). This resulted in respectively 376 and 36 studies that addressed dispersal of alien plants for endo- and ectozoochory. Addition of the term TS = (aquatic OR wetland OR freshwater OR riparian) reduced the publication counts to respectively 16 and 6, indicating that a low percentage of all studies involved aquatic habitats.

Examples of interference with endozoochory notably included cases with terrestrial birds, such as silvereye *Zosterops lateralis*, Japanese white-eye *Zosterops japonicas*, southern cassowary *Casuarius casuarius* and emus *Dromaius novaehollandia* (Stanley and Lill, 2002; Bradford et al., 2008; Kawakami et al., 2009; Calvino-Cancela, 2011) or mammals such as deer, boar and cattle (Bartuszevige and Endress, 2008; Vignolio and Fernández, 2010; Dovrat et al., 2012). I found few studies on potential interference for other major disperser animals such as bats, and no studies on fish or reptiles. Examples of externally dispersed alien diaspores (and although not explicitly mentioned thus potentially interfering with native diaspore dispersal) mostly studied mammals such as bison *Bison bison*, wild boar *Sus scrofa* and cattle (Constible et al., 2005; Dovrat et al., 2012; Chuong et al., 2016). For ectozoochory, there is a need for more studies on other taxa than mammals.

A more detailed search to specifically examine interference in aquatic ecosystems in Web of Science and Google Scholar, and a cross-reference search on recent reviews (Reynolds et al., 2015; Green, 2016), resulted in a total of 8 publications on endozoochory (Table 1) and 5 on ectozoochory (Table 2) reporting data interpretable for interference by alien species. The need for this more extensive search, however, revealed a problem. Many studies on zoochory—and therefore potentially reporting interference—do not report the native or alien status of dispersed diaspores (e.g., Viviansmith and Stiles, 1994). Without specific knowledge on the exact study systems and dispersed taxa, potential interference of alien plants with zoochory is easily overlooked by readers that lack system-specific knowledge - and impossible to detect in searches on alien species. I therefore recommend future studies on zoochory to report the alien/native status of dispersed taxa more explicitly, as this will greatly advance our understanding of the scale of potential interference across ecosystems and taxa.

**Table 1:** Examples of field studies reporting plant diaspores in sampled feces of aquatic animals for both native and invasive species within single sampling events.

**Vector**	**Publication**	**Location**	**Native species**	**Alien species**	**Disperser animal**	**Native diaspores**	**Alien diaspores**	**% alien**	***n***
Waterbirds	Powers et al., 1978	LA, USA	Table 2^a^	*Cyperus iria, Eleocharis obtusa, Echinochloa colonum, Fimbristylis miliacea*	7 species of waterfowl^b^	1047	110	10	51
Waterbirds	Sanchez et al., 2006	Odiel marshes, Spain	*Arthrocnemum macrostachyum*	*Mesembryanthemum nodiflorum, Sonchus oleraceus*	Black-tailed Godwit *Limosa limosa*	5	1	17	66
Waterbirds	Sanchez et al., 2006	Odiel marshes, Spain	*A. macrostachyum*	*M. nodiflorum, S. oleraceus*	Redshank *Tringa totanus*	5	30	86	86
Waterbirds	Green et al., 2008	NSW, Australia	Table 3^a^	*Ranunculus scleratus, Medicago polymorpha, Polygonum arenasturm*	Grey teal *Anas gracilis*	163	12	7	30
Waterbirds	Green et al., 2008	NSW, Australia	Table 3^a^	*R. scleratus, M. polymorpha, P. arenasturm*	Black swan *Cygnus atratus*	11	5	31	20
Waterbirds	Green et al., 2008	NSW, Australia	Table 3^a^	*R. scleratus, M. polymorpha, P. arenasturm*	Eurasian coot *Fulica atra*	18	0	0	20
Waterbirds	Green et al., 2008	NSW, Australia	Table 3^a^	*R. scleratus, M. polymorpha, P. arenasturm*	Australian pelican *Pelecanus conspicillatus*	116	0	0	1
Waterbirds	Brochet et al., 2009	Camargue, France	Table 2^a^	*Heteranthera reniformis*	Eurasian teal *Anas crecca*	11332	1186	9	42
Waterbirds	Best and Arcese, 2009	BC, Canada	*Myosurus minimus, Epilobium ciliatum*	*Poa annua, Aira praecox, Silene gallica*	Canada goose *Branta canadensis*	5	20	80	314^c^
Waterbirds	Brochet et al., 2010	Camargue, France	Table 3^a^	*Ludwigia peploides, Paspalum distichm, H. reniformis, H. limosa*	Eurasian teal *A. crecca*	893	9	1	366
Waterbirds	Raulings et al., 2011	VIC, Australia	Table 2^a^	*Cotula cioronopifolia, Lactuca serriola, Sonchus oleraceus, Trifolium glomeratum*	Pacific black duck *Anas superciliosa*	25	5	17	49
Waterbirds	Raulings et al., 2011	VIC, Australia	Table 2^a^	*C. cioronopifolia, L. serriola, S. oleraceus, T. glomeratum*	Chestnut teal *Anas castanea*	11	8	42	52
Waterbirds	Raulings et al., 2011	VIC, Australia	Table 2^a^	*C. cioronopifolia, L. serriola, S. oleraceus, T. glomeratum*	Grey teal *A. gracilis*	19	7	27	46
Waterbirds	Reynolds and Cumming, 2016	South Africa	Table S3^a^	Table S3^a^	Egyptian goose *Alopochen aegyptiaca*	6	736	99	145
Waterbirds	Reynolds and Cumming, 2016	South Africa	Table S3^a^	Table S3^a^	Red-billed teal *Anas erythrorhyncha*	8	4	33	35
Waterbirds	Reynolds and Cumming, 2016	South Africa	Table S3^a^	Table S3^a^	White-faced duck *Dendrocygna viduata*	0	6	100	8
Waterbirds	Reynolds and Cumming, 2016	South Africa	Table S3^a^	Table S3^a^	Yellow-billed duck *Anas undulata*	9	2	18	60
Waterbirds	Reynolds and Cumming, 2016	South Africa	Table S3^a^	Table S3^a^	Spur-winged goose *Plectropterus gambensis*	2	2	50	30
Waterbirds	Reynolds and Cumming, 2016	South Africa	Table S3^a^	Table S3^a^	Cape shoveler *Anas smithii*	7	60	90	35

*The number of fecal samples investigated is denoted by n*.

a *Refers to the table in the source publication*.

b *Reported and analyzed as if one species*.

c *Grams of dried feces*.

**Table 2:** Examples of field studies reporting diaspores attached to aquatic animals for both native and alien species within single sampling events.

**Vector**	**Publication**	**Location**	**Native species**	**Alien species**	**Disperser animal**	**Native diaspores**	**Alien diaspores**	**% alien**	***n***
Waterbirds	Viviansmith and Stiles, 1994	NJ, USA	Table 1^a^	*Sonchus asper*	Brant *B. bernicla*	53	2	4	24
Waterbirds	Viviansmith and Stiles, 1994	NJ, USA	Table 1^a^	*S. asper*	Bufflehead *Bucephala albeola*	10	2	17	6
Waterbirds	Viviansmith and Stiles, 1994	NJ, USA	Table 1^a^	*S. asper*	American Black duck *Anas rubripers*	12	0	0	4
Waterbirds	Viviansmith and Stiles, 1994	NJ, USA	Table 1^a^	*S. asper*	Red-breasted Merganser *Mergus serrator*	10	0	0	2
Waterbirds	Tøttrup et al., 2010	Norway and Finland	None	*Fistulobalanus pallidus*	Lesser black-backed gull *Larus fuscus*	0	>41	100	7
Waterbirds	Raulings et al., 2011	VIC, Australia	*Conyza bonariensis, Lachnagrostis filiformis*	*Trifolium cf. glomeratum, Plantago coronopus, Senecio glomeratus*	Chestnut teal *A. castanea*	7	1	13	22
Waterbirds	Raulings et al., 2011	VIC, Australia	*Conyza bonariensis, Lachnagrostis filiformis*	*T. cf. glomeratum, P. coronopus, S. glomeratus*	Pacific Black Duck *A. superciliosa*	1	2	67	3
Seabirds	Aoyama et al., 2012	Ogasawara Islands, Japan	*Sporobolus diander*	*Chloris barbata*	Black-footed albatross *Phoebastria nigripes*	1	7	88	41
Seabirds	Aoyama et al., 2012	Ogasawara Islands, Japan	*Digitaria pruriens*	*Cenchrus echinatus, Boerhavia diffusa*	Bulwer's petrel *Bulweria bulwerii*	1	11	92	45
Seabirds	Aoyama et al., 2012	Ogasawara Islands, Japan	None	*C. echinatus, C. barbata*	Wedge-tailed shearwater *Puffinus pacificus*	0	17	100	45
Seabirds	Aoyama et al., 2012	Ogasawara Islands, Japan	None	*C. barbata, B. diffusa*	Brown booby *Sula leucogaster*	0	12	100	29
Waterbirds	Reynolds and Cumming, 2016	South Africa	Table S2^a^	Table S2^a^	Egyptian goose *Alopochen aegyptiaca*	20	66	77	194
Waterbirds	Reynolds and Cumming, 2016	South Africa	Table S2^a^	Table S2^a^	Red-billed teal *Anas erythrorhyncha*	7	23	77	49
Waterbirds	Reynolds and Cumming, 2016	South Africa	Table S2^a^	Table S2^a^	White-faced duck *Dendrocygna viduata*	3	2	40	8
Waterbirds	Reynolds and Cumming, 2016	South Africa	Table S2^a^	Table S2^a^	Yellow-billed duck *Anas undulata*	50	58	54	141

*The number of sampled animals is denoted by n*.

a *Refers to the table in the source publication*.

All aquatic studies jointly reported 34 cases of potential interference by alien plants with dispersal of native diaspores—by native disperser animals—and in aquatic ecosystems (Tables 1, 2). The percentages of alien species in feces or attached to single disperser animals ranged from 0 to 100% of all diaspores being alien, which was the case for both endozoochory and ectozoochory (Tables 1, 2). The mean percentage of alien species was 38% for endozoochory (*n* = 1142 fecal samples) and 55% for ectozoochory (*n* = 620 investigated animals). In 5 of the 19 reports on endozoochory and 8 of the 15 reports of ectozoochory more alien than native diaspores were dispersed. However, these numbers should be interpreted with caution because variation among geographic locations and studied species was large, and studies reporting only dispersal of native diaspores were not included.

The presented cases illustrate that patterns of alien interference thus far primarily described in terrestrial ecosystems may also apply to aquatic ecosystems. Future research should focus on under-examined disperser animals like fish, bats and reptiles (Horn et al., 2011; Jordaan et al., 2011; Platt et al., 2013). Studying broader taxonomic ranges of both diaspores and disperser animals can identify which native communities are most susceptible to interference by alien species, test for differences between interference in species rich and species poor communities, analyze latitudinal trends, or detect new suitable model systems in which interference with endo- and ectozoochory can be studied simultaneously. Furthermore, disperser diets and adhering diaspores could be compared between situations from before and after invasions in the same system if more data are available (Gosper et al., 2006). Levels of alien interference could be contrasted between endo- and ectozoochory and among different disperser species in the same community; for instance by supplementing field or laboratory setups simultaneously with externally and internally dispersing alien diaspores.

## Positive and negative effects of alien plants on zoochory of native plants

The fact that alien plants can interfere with zoochory of native plants in terrestrial and aquatic ecosystems raises questions about the magnitude and the directions of these effects. Especially directions are crucial to understand in the light of possible control or eradication of alien species for conservation. This section explores possible negative and positive effects of alien species on endo- and ectozoochory of native species.

Introductions of alien species are commonly associated with loss of native species and deteriorating ecosystems, because alien species are thought to directly outcompete native species or indirectly affect abiotic conditions (Morales and Traveset, 2009; Havel et al., 2015; Gilioli et al., 2017). However, alien species that successfully integrate into resident communities can also stabilize networks by increasing network nestedness (Bascompte et al., 2003; Traveset et al., 2013), boost productivity by increasing overall species richness (Cardinale et al., 2006) or provide new ecosystem services to native species (Gleditsch, 2017). Alien species can benefit disturbed communities (Lugo, 2004) or compensate for the loss of native species (Kawakami et al., 2009). I here discuss the possibility that alien species stimulate zoochory of native species by exploring two possible mechanisms: alien species may affect the abundances or behavior of native disperser animals, or may facilitate the arrival of new alien disperser species.

The first possible mechanism assumes that establishment of alien plants can increase the local densities of native disperser animals already present in the community. The attractiveness of an individual plant for disperser animals relies partly on its surrounding plant species (Carlo, 2005), which is a well-established phenomenon in pollination ecology (e.g., Bruckman and Campbell, 2016). Because most animals actively track fluctuations in resources across the landscape (Saracco et al., 2004; Cameron and Bayne, 2012), productive alien plant species at high densities can increase abundances of disperser species or alter their movement behavior. Reports of this scenario are still rare, but an elegant example involves two invasive *Lonicera* species that attract native birds to their fruits, which increases removal of nearby native fruits by one-third (Gleditsch and Carlo, 2011). Hence, productive alien plants can increase densities of local native dispersers that can transport native diaspores either internally or externally. We can expect alien species that provide attractive new resources to native dispersers—and are therefore often dispersed via endozoochory—to have a greater potential to influence abundances and behavior of native disperser animals than alien plants primarily dispersed by passive attachment to animals.

The second possible mechanism is through the attraction of new alien disperser animal species. Alien plants can facilitate establishment of alien animal species relying on newly provided resources such as food, refugia against predators, or nesting substrate (Chiba, 2010; Schlossberg and King, 2010; Schlaepfer et al., 2011; Nelson et al., 2017). Hence, new disperser animals may be attracted by alien plants, which could also benefit either endo- or ectozoochory of native plant species. However, in case of endozoochory, alien animals often forage primarily on alien fruits (Chimera and Drake, 2010; Garcia et al., 2014; Pejchar, 2015; Schor et al., 2015), and their movement after ingestion can deviate from that of native dispersers. If alien disperser animals compete with native disperser animals, the potential for effective and successful endozoochory to suitable habitat may actually decrease. For ectozoochory, addition of new disperser species with differing behavior from native dispersers may vary from facilitation of range expansions to transport of diaspores to unsuitable habitats because of unfitting movement behavior. The overall effect of new alien dispersers on native plants will therefore largely vary among species networks and dispersal mechanisms.

The above examples suggest that alien plant species can—in some systems—actually positively affect endo- and ectozoochory of native species via trophic interactions (e.g., Gleditsch and Carlo, 2011). However, they also indicate that this is still a little explored research direction. Fruitful directions for future studies are therefore to (1) experimentally attract disperser animals with artificial diaspores to mimic attraction by alien species (Galetti et al., 2003) in the field or in laboratory setups, and monitor the effects on zoochory of native plants; (2) use large, long-term datasets on species interactions that are currently becoming available (Bello et al., 2017) to compare species interactions and networks between before and after invasions; (3) further explore the effects of timing of fruit set on species interactions. Competition of aliens with natives plants is strongly related to the timing of fruit sets (Buckley et al., 2006) and uncoupled fruiting seasons may lower possible competition for dispersers, while longer fruiting seasons may have positive effects on disperser species. These possible scenarios could be contrasted using theoretical modeling or field data. Finally, (4) future studies can extract directions from the strong analogies with pollination ecology (e.g., Richardson et al., 2000a; Traveset and Richardson, 2006; Bjerknes et al., 2007; Seifan et al., 2014; Bruckman and Campbell, 2016).

## Integrative conclusions

This study explored interference of alien plant species with zoochory of native plants, and concludes that (1) although the phenomenon has been primarily studied for endozoochory by frugivorous birds and mammals in terrestrial ecosystems, alien species may also interfere with ectozoochory and this warrants further studying; (2) interference of alien species with zoochory can similarly be found in aquatic ecosystems; (3) alien plant species can also provide resources such as food or nesting habitat to animals, which can increase densities of native disperser animals or attract new disperser animal species. Through these mechanisms alien plants can also positively affect dispersal of native plants, which warrants further studying. This study illustrates that the impacts of alien plant species on native plant species, whether positive or negative, can vary among native plant species relying on different dispersal mechanisms. Understanding species interactions is crucial for effective conservation, especially in invaded ecosystems.

## Author contributions

CvL designed the study, collected and analyzed the data, and wrote the manuscript.

### Conflict of interest statement

The author declares that the research was conducted in the absence of any commercial or financial relationships that could be construed as a potential conflict of interest.
